# Back Propagation Neural Network Model for Predicting the Performance of Immobilized Cell Biofilters Handling Gas-Phase Hydrogen Sulphide and Ammonia

**DOI:** 10.1155/2013/463401

**Published:** 2013-11-07

**Authors:** Eldon R. Rene, M. Estefanía López, Jung Hoon Kim, Hung Suck Park

**Affiliations:** ^1^Core Group Pollution Prevention and Resource Recovery, Department of Environmental Engineering and Water Technology, UNESCO-IHE Institute for Water Education, P.O. Box 3015, 2601 DA Delft, The Netherlands; ^2^Chemical Engineering Laboratory, Faculty of Sciences, University of La Coruña, Rúa da Fraga 10, 15008 La Coruña, Spain; ^3^Department of Civil and Environmental Engineering, University of Ulsan, P.O. Box 18, Ulsan 680-749, Republic of Korea

## Abstract

Lab scale studies were conducted to evaluate the performance of two simultaneously operated immobilized cell biofilters (ICBs) for removing hydrogen sulphide (H_2_S) and ammonia (NH_3_) from gas phase. The removal efficiencies (REs) of the biofilter treating H_2_S varied from 50 to 100% at inlet loading rates (ILRs) varying up to 13 g H_2_S/m^3^ *·*h, while the NH_3_ biofilter showed REs ranging from 60 to 100% at ILRs varying between 0.5 and 5.5 g NH_3_/m^3^ *·*h. An application of the back propagation neural network (BPNN) to predict the performance parameter, namely, RE (%) using this experimental data is presented in this paper. The input parameters to the network were unit flow (per min) and inlet concentrations (ppmv), respectively. The accuracy of BPNN-based model predictions were evaluated by providing the trained network topology with a test dataset and also by calculating the regression coefficient (*R* ^2^) values. The results from this predictive modeling work showed that BPNNs were able to predict the RE of both the ICBs efficiently.

## 1. Introduction

A typical landfill gas consists of methane (45–60% v/v), carbon dioxide (40–60% v/v), and other compounds that include nitrogen, oxygen, sulphides, ammonia, carbon monoxides, and trace constituents. The amount of landfill gas generated is proportional to the amount of organic waste present and is produced by the bacteria during decomposition. These gases can easily move through the landfill surface to the ambient air and then to the community with the wind. The sulphur compounds (mercaptans and hydrogen sulphide) are the main contributors to the persisting odor problem from landfills, which are also considered toxic [1]. On the other hand, ammonia is both a potentially toxic product of refuse degradation and an essential nutrient for the bacteria responsible for this. The presence of these pollutants in the atmosphere has shown to cause significant damage to both human health and natural environment [2, 3]. In South Korea, there are a large number of landfills that do not incorporate suitable strategies to prevent these emissions from reaching the nearby community. Hence, there arises potential necessity to adapt worthy control techniques for effectively removing these emissions from landfills.

Biological treatment systems such as biofilters, and biotrickling filters have been demonstrated for several decades to be a cost effective technology for the treatment of waste gases containing low concentrations of contaminants at large flow rates [4–6]. The high removal efficiencies (REs) achieved along with its uncomplicated and flexible design, low operational, and maintenance costs edges biofilters over other biological treatment techniques such as biotrickling filters and bioscrubbers [7–11]. Biofilters can effectively remove H_2_S and NH_3_ emissions from waste-gas streams using a bed of biologically active material such as compost, peat, and wood bark. Belatedly, immobilization of microbes in suitable support matrix such as alginate beads or suitable polymeric materials has gained popularity in the research domain of biofiltration. The principal advantages of adopting immobilization techniques in biofiltration is to provide high cell concentrations, improve genetic stability, protecting the microbes from shear damage, and to enhance favorable microenvironment for microbes (nutrient gradients and pH). *Pseudomonas putida* CH11 was tested for the removal of H_2_S in both batch and continuous systems (pH: 6.0–8.0), yielding maximum removal rate and saturation constant values of *V*_*m*_ = 1.36 g S/day*·*kg dry bead and *K*_*s*_ = 45.9 ppm, respectively [12]. A biofilter inoculated with *Nitrosomonas europaea* was used to remove gaseous ammonia, in the concentration range of 10 or 20 ppm showed 99% RE after 4 days of operation [13]. The effects of operational factors such as retention time, temperature, and inlet concentration on the performance of a biofilter packed with *Thiobacillus thioparus* immobilized with Ca-alginate pellets were evaluated and found to have an optimal S-loading of 25 g/m^3^·h, in order to achieve high removal of that compound [14]. For the treatment of landfill gas containing H_2_S and NH_3_, they can be easily treated by two immobilized cell biofilters (ICB) with different microorganisms in series or single ICB column with mixed microorganisms, as shown in our previous studies [2, 3].

Traditionally, the performance of biofilters has been modeled/predicted using process-based models that are based on mass balance principles, simple reaction kinetics, and a plug flow of air stream [15–18]. The main advantages of these process models are that, they are anchored on the underlying physical process and the results obtained from these process models generally provide a good understanding and interpretation of the system. However, this depends on numerous model parameters and obligates selective information on specific growth rate of microbes, biofilm thickness and density, values of diffusivity, partition, yield and distribution coefficients, intrinsic adsorption, and so forth [19–21]. The accurate estimation of some of these parameters requires elaborated technical facilities and expertise, the absence of which hinders the preciseness of the model and limits the application and reliability of the model. 

An alternate modeling procedure consists of a data driven approach wherein the principles of artificial intelligence (AI) is applied with the help of neural networks [22]. The concept of neural network modeling has widespread applications in the field of applied science and engineering. An ANN-based model was developed to simulate different types of biomass for a gasification process and it was demonstrated that the model predicted profiles matched closely to the experimental values [23]. ANN model based on wavelet packet decomposition, entropy, and neural networks was formulated to predict the long-term performance of a wastewater treatment plant [24]. A 3-layered neural network with the standard back propagation algorithm was used in their study and the authors reported that the model was able to predict plant performance better. Recently, an ANN-based software was developed to predict thermal power plant effluent temperature that could help in optimizing load generation among different power generation units and this software demonstrated its ability to predict the canal temperature over the normal operating range with high accuracy [25]. With respect to the application of ANN for optimization purposes, ANN and genetic algorithm-based techniques were combined together to optimize media constituents, in order to enhance lipase production by soil microbes [26]. The results from their study showed that ANN-based model was able to predict the system behavior clearly showing lipolytic activity of 7.69 U/mL. It has been shown quite recently that the performance of biofilters and/-or biotrickling filters can be predicted from prior estimation of easily measurable operational parameters using ANNs [27–30]. In our previous studies, ANN-based predictive approach was proposed to model the performance of individually operated ICBs for H_2_S and NH_3_ removal, respectively [31, 32]. The outputs of the model were RE and EC, respectively, while the input parameters to the model were inlet concentration, loading rate, flow rate, and filter-bed pressure drop, respectively. The results for the H_2_S operated ICB showed that a multilayer network (4-4-2) with back propagation algorithm was able to predict the ICB performance effectively with a *R* ^2^ values of 0.9157 and 0.9965 for removal efficiency and elimination capacity, respectively [31]. Similarly, for the ICB treating NH_3_, multilayer network (4-4-2) with error back propagation predicted the RE and EC with *R* ^2^ values of 0.9825 and 0.9982, respectively [32].

The objectives of this research work were to experimentally evaluate the collective performance of two biofilters treating H_2_S and NH_3_ and to predict the ICBs performance parameter, namely RE, using one back propagation neural network (BPNN). Experiment data collected from our previous studies [2, 3] were thus integrated for predicting the RE profiles of H_2_S and NH_3_ using the BPNN. The input parameters to the model were unit flow (gas-flow rate/volume) and inlet concentrations, while the output parameter was the RE of the ICBs. After model development, the input parameters were subjected to sensitivity analysis in order to understand their effects on the RE profiles.

## 2. The Simple Back Propagation Neural Network Approach

Multilayer perceptron (MLP) using the back propagation algorithm [26, 33] is the most widely used neural network for forecasting/prediction purposes [34–36]. Neural networks acquire their name from the simple processing units in the brain called neurons which are interconnected by a network that transmits signals between them. These can be thought of as a black box device that accepts inputs and produces a desired output. MLP generally consists of three layers; an input layer, a hidden layer, and an output layer [36]. Each layer consists of neurons which are connected to the neurons in the previous and flowing layers by connection weights (*W*_*ij*_). These weights are adjusted according to the mapping capability of the trained network. An additional bias term (*θ*_*j*_) is provided to introduce a threshold for the activation of neurons. The input data (*X*_*i*_) is presented to the network through the input layer, which is then passed to the hidden layer along with the weights. The weighted output (*X*_*i*_ *W*_*ij*_) is then summed and added to a threshold to produce the neuron input (*I*_*j*_) in the output layer that can be represented by

(1) $$\begin{array}{c} \;I_{j}=\;\sum_{i,j=1}^{i,j=8}W_{ij}X_{i}+\theta_{j}. \end{array}$$

This neuron input passes through an activation function *f*(*I*_*j*_) to produce the desired output *Y*_*j*_. The most commonly used activation function is the logistic sigmoid function which takes the form;

(2) $$\begin{array}{c} f\left(I_{j}\right)=\frac{1}{1+e^{-I_{j}}}. \end{array}$$

## 3. Modeling Methodology

### 3.1. Model Input-Outputs and Data Division

A combined neural network-based predictive model was developed for the two biofilters using unit flow (*X*_1_) and inlet concentration (*X*_2_) as the model inputs and removal efficiency (*Y*_1_) as the output. The experimental data was divided into training (*N*_*Tr*⁡_, 75%) and test data (*N*_Te_, 25%). The test data was set aside during network training and was only used for evaluating the predictive potentiality of the trained network. The basic statistics of the variables for the training and test matrix is shown in Tables 1 and 2, respectively.

### 3.2. Error Evaluation

 The closeness of prediction between the experimental and model predicted outputs were evaluated by computing the determination coefficient values as shown below [27];

(3) $$\begin{array}{c} R^{2}={[\frac{\sum_{i=1}^{N}\left(Y_{{\text{model}}_{i}}-\overset{-}{Y_{\text{model}}}\right)\left(Y_{{\text{observed}}_{i}}-\overset{-}{Y_{\text{observed}}}\right)\;\;}{\left(N-1\right)S_{Y_{\text{model}}}S_{Y_{\text{observed}\;}}}]}^{2}, \end{array}$$

where *Y*_model_*i*__—predictions made by the model, *Y*_observed_*i*__—observed true values from experiments, *N*—number of cases analyzed, $\bar{Y}$—average value, and *S*_*Y*_—standard deviations.

### 3.3. Data Preprocessing and Randomization

Experimental data collected from the biofilters during the 67 × 2 days (2 denotes the two biofilters) of continuous operation was randomized to obtain a spatial distribution of the data, which accounts for both steady state and transient (or) quasi-steady-state operations. The data was also normalized and scaled to the range of 0 to 1 using (4), so as to suit the transfer function in the hidden (sigmoid) and output layer (linear)

(4) $$\begin{array}{c} \hat{X}=\frac{X-X_{\min}}{X_{\max}-X_{\min}}, \end{array}$$

where $\hat{X}$ is the normalized value and *X*_min⁡_ and *X*_max⁡_ are the minimum and maximum values of *X* respectively.

### 3.4. Network Parameters

The internal parameters of the back propagation network, namely, epoch size, error function, learning rate (*η*), momentum term (*α*), training cycle (*T*_c_), and transfer function are to be appropriately selected to obtain the best network architecture that gives high predictions for the performance variables. In this study, the number of neurons in the input layer (*N*_*I*_ = 2) and output layer (*N*_*O*_ = 1) were chosen based on the number of input and output variables to the network. A detailed study on the effect of internal network parameters on the performance of back propagation networks [37] and the procedure involved in selecting the best network topology has been described elsewhere [34, 35]. However, in most instances, literature suggests the use of a trial and error approach where the performance goal is set by the user. In this study, the best values of the network parameters were chosen by carrying out simulations using a trial and error approach. The best network was chosen based on the maximum predictability of the network for the test data by analyzing the determination coefficient values. 

### 3.5. Software Used

BPNN-based predictive modeling was carried out using the shareware version of the neural network and multivariable statistical modeling software, NNMODEL (Version 1.4, Neural Fusion, NY, USA).

### 3.6. Experimental Materials and Methods

The details of the experimental strategy adopted, inoculum, media composition, preparation of immobilized packing media, experimental setup, ICB operation, and analytical techniques for data collection have been detailed in our previously published work [2, 3]. 

## 4. Results and Discussions

### 4.1. Experimental

**Figure 1:**
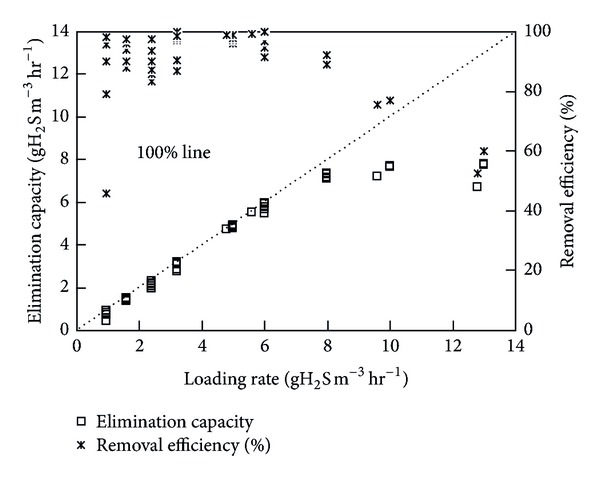
Effect of inlet loading rate on the elimination capacity and removal efficiency profiles of the immobilized cell biofilter handling H_2_S vapors (More details can be seen in [3]).

**Figure 2:**
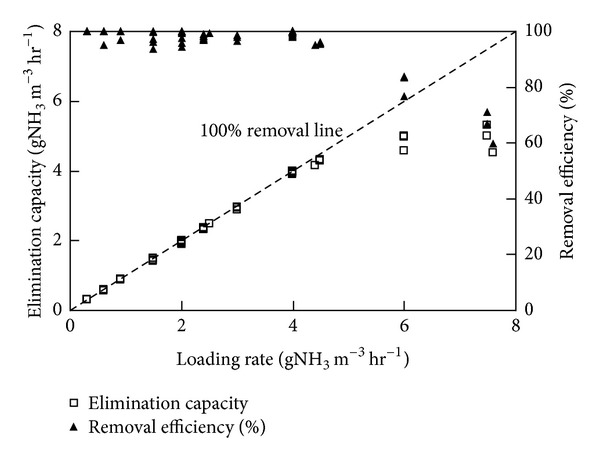
Effect of inlet loading rate on the elimination capacity and removal efficiency profiles of the immobilized cell biofilter handling NH_3_ vapors (More details can be seen in [2]).

The initial inlet loading rates (ILRs) to both the biofilters were sufficiently low (<1 gH_2_S (or) NH_3_/m^3^·h), that allowed the immobilized microbes to acclimatize themselves to the vapor phase pollutant. Once acclimatized (high removal, RE > 95%), the ICBs were subjected to a step-wise increase in ILRs by gradually varying the inlet concentration of either H_2_S or NH_3_ to the ICBs. During every step increase in the ILR, it was observed that the biofilter took about 2 to 4 d to adapt to the new concentration and reached a new steady state value shortly. Initially, when the loading rates were <1 g/m^3^·h, the RE increased gradually from 45 to ~100%, which indicated good activity of the immobilized cells to treat these pollutants. The removal profiles and EC achieved for both the biofilters during the entire operational steps are shown in Figures 1 and 2, as a function of the ILRs. For the ICB treating H_2_S vapors, the input was changed in 7 steps up to a ILR of 8 gH_2_S/m^3^·h, during which the RE remained constant at 82%. It has been shown that H_2_S metabolism by heterotrophic sulphur oxidizing bacteria is a detoxification process and high inlet concentrations have often been reported to decrease the H_2_S removal efficiency [15]. The EC profiles were almost linear till an ILR of 8 gH_2_S/m^3^·h, which indicates that the biofilter performed with 100% efficiency till this critical load [9]. For the ICB treating NH_3_, it is evident that the RE was nearly >95% up to a ILR of 4.5 gNH_3_/m^3^·h. However, when the ILR was increased significantly by varying both the concentration and flow rate to values as high as 7.5 gNH_3_/m^3^·h, a noticeable decrease in the RE values from 100% to ~60% was observed. The critical NH_3_ loading rate to the biofilter was considered as 4.5 gNH_3_/m^3^·h. Pressure drop values were sufficiently low during the operational time for both of the ICBs (0.1–1.7 cm of H_2_O) and did not cause any significant operational problem. These values of pressure drop are within the safe operational range suggested for full-scale biofilter operation [2, 4, 9]. 

### 4.2. BPNN Modeling

#### 4.2.1. Network Architecture

Artificial neural network-based models requires the best combinations of network parameters such as training cycle (*T*_*c*_), neurons in the input (*N*_*I*_), hidden (*N*_*H*_) and output layer (*N*_*O*_), learning rate (*η*), momentum term (*α*), and a good algorithm for the predictions to be accurate [2, 3, 36]. In this study, the models for predicting the RE of ICBs were trained and tested adequately with the experimental data and evaluated by the determination coefficient values between the measured and predicted outputs from the network.  Table 3 shows the different network parameters used for training the network. The algorithm used for training in this study was the standard back error propagation (BEP) algorithm, which has potentially shown to exhibit high capability in predicting process variables [38, 39]. The model was trained using different combinations of these parameters so as to achieve maximum determination coefficient values (target value = 1, i.e., 100% correlation between measured and predicted variables). This was achieved by a vigorous trial and error approach by keeping some training parameters constant and by slowly moving the other parameters over a wide range of values, as suggested in some previous works [26, 34, 35]. A trial and error approach was followed in this study to determine the best network topology and the effect of internal network parameters due to the following reasons: (i) there were several parameters whose values had to be varied from low to high values (example: learning rate from 0.1–1; momentum term from 0.1–1), by keeping other parameters constant, and (ii) although several literatures have suggested different heuristic rules for selecting the (best) parameters, adequate training of the network always remains a key issue during ANN modeling, as this largely depends on the complexity of the process, the quality of data obtained, and the nature of interpretation done by the user. In this study, the following observations were made during training: (i) increasing the number of neurons in the hidden layer from 2 to 8 did not significantly increase the *R* ^*2*^ values, and the value of 2 was finally chosen, (ii) the training cycle appears to have a tremendous influence in increasing the *R* ^*2*^ values and it was observed that the model predictions were high and significant when the training cycle was set to 40,000, (iii) similarly, high learning rates seem to invariably increase the prediction efficiency, and (iv) low values of momentum term showed* R* ^*2*^ values greater than 0.84 in the test data during the predictions of RE. The *R* ^*2*^ values during training and testing were 0.8716 and 0.8484, respectively. Thus, only about 13–16% of the total deviations could not be explained by the model for predicting the combined removal efficiency profiles in the ICBs. The best network architecture for this combined model is 2-2-1. The results from this study indicate high learning rate (*η*-0.9), low momentum term (*α*-0.3), and a training cycle of 40,000 with 2 neurons in the hidden layer (*N*_*H*_) are favorable values of the internal network parameters.

#### 4.2.2. Predictive Potentiality of the Model

**Figure 3:**
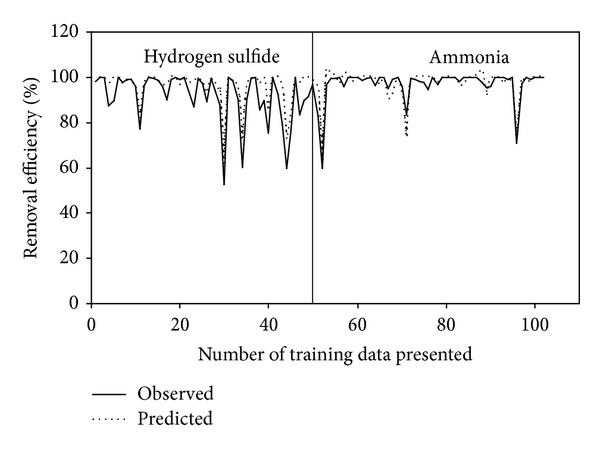
Observed and BPNN predicted values of removal efficiency profiles during training.

**Figure 4:**
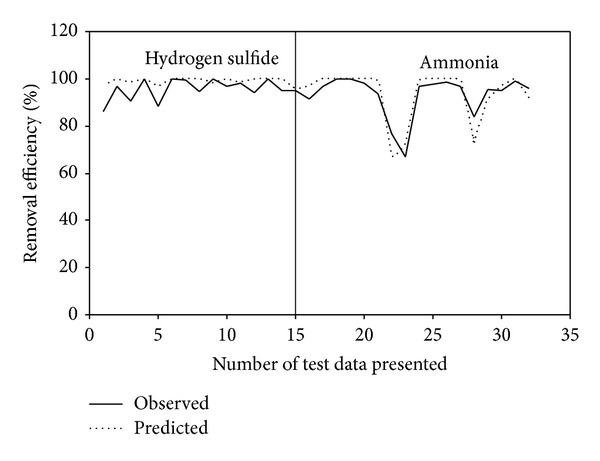
Observed and BPNN predicted values of removal efficiency profiles during testing.

The performance parameter of the ICB treating H_2_S and NH_3_, namely RE, for the training and test data is shown in Figures 3 and 4, respectively. It can be observed that some of the data points for both H_2_S and NH_3_ were not predicted properly by the BPNN model, thus leading to large errors, ~13%. This could be due to the quasi-steady-state attained in the two ICBs, when the loading rate was step increased from one level to another. During this stage, the biofilter took some time (3 to 4 d) to adjust itself to the new concentration, thereby achieving steady state removals [2, 3]. Moreover, corroborating these deviations is the less critical load in the NH_3_ biofilter (4.5 g/m^3^·h) in comparison to the H_2_S biofilter (8 g/m^3^·h). This decrease in critical loads and corresponding removal profiles would have caused an impact in the networks generalization pattern while predicting the performance parameters, a pattern that has been often reported in biofilter and biotrickling filter operations [7, 9, 12]. However, the BPNN-based model showed good predictive ability for performance variables as seen from the closeness of the fit between the experimental and predicted observations. 

**Figure 5:**
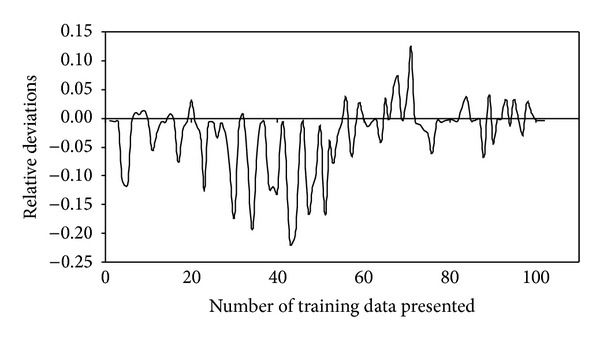
Relative deviations observed during model predictions for removal efficiency in the training data set.

**Figure 6:**
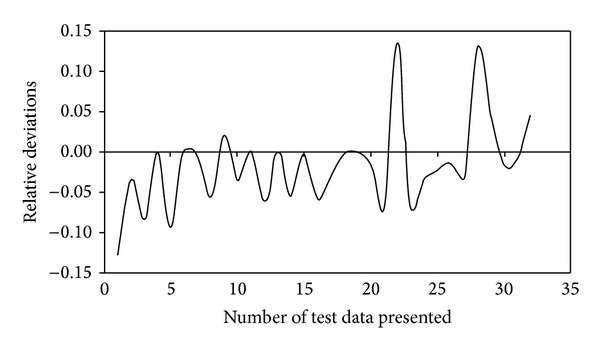
Relative deviations observed during model predictions for removal efficiency in the test data set.

Anew, the predictive capacity of the network was also evaluated in terms of its relative deviation, that is, (RE_exp⁡_− RE_pred_)/RE_exp⁡_. These deviations for removal efficiency predicted by model during network training and testing are shown in Figures 5 and 6, respectively. The relative deviations are more significant, that is, >15% in some cases, which can be attributed to the change in load to the ICBs. This could be further explained by the EC profiles showed in Figures 1 and 2, respectively. For higher initial concentration and higher flow rate (high loading rates), the EC of the filter bed increased at a slower rate, becoming nearly constant at inlet loads beyond 8 g H_2_S/m^3^·h and 4.5 g NH_3_/m^3^·h, respectively. This phenomenon could be possibly due to the reaction and diffusion limitation steps as explained by Ottengraf [38], or by any one of the following mechanism; (i) smaller pore sizes in the media could restrict the accessibility of nutrients on the pore surface by the microorganisms, while at large pore size the specific surface area may be the limiting factor, (ii) at high cell densities, intra particle pore diffusion limitations have shown to play a significant role in reducing the elimination capacities, and (iii) microenvironmental conditions inside the encapsulated media could also vary with position and affect the physiology of the cells. The decline in RE at high loading rates could also be attributed to some complex mechanisms associated with the removal profiles in the immobilized media, where the waste air is first scrubbed and/-or absorbed in the liquid biofilm and then oxidized by the microorganisms. 

**Figure 7:**
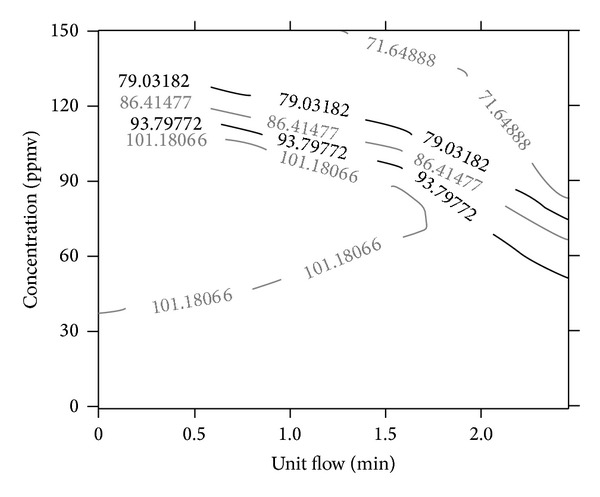
Contour plot showing the operating regime to achieve greater than 93.7% removal efficiency.

The weights and bias terms between the hidden layer connections [39] obtained after network training is given in Table 4. In order to evaluate the significant effect of the input parameters on the developed model, a sensitivity analysis was carried out by estimating the Absolute Average Sensitivity (AAS). The sensitivity is calculated by summing the changes in the output variables caused by moving the input variables by a small amount over the entire training set. The AAS is the absolute values of the change in the input [40]. The computed AAS value on different input parameters for model is shown in Table 5. Unit flow (0.5628) appears to have a more significant effect in predicting RE profiles in the ICBs than the concentration term. The results from this analysis reveal the degree of relevance of the input parameters to the outputs.  Figure 7 shows the contour plot of RE, as a function of the concentration and unit flow for the ICB. This contour plot can be interpreted as follows: RE > 93.7% can be consistently maintained in the ICB, if the following condition is met: inlet H_2_S or NH_3_ concentration is constantly maintained at less than 120 ppmv, at a unit flow of 2 per min.

The predictive ability of the proposed model using the concepts of artificial intelligence and the back propagation algorithm was high and significant, as ascertained from the *R* ^*2*^ value between the measured and predicted outputs in the training and test data for predicting RE of the ICB. This work could enable researches to extend and intensify research in BPNNs for evaluating pilot scale ICBs, besides helping in optimizing their state variables. For practical applications, ANNs can be used for real-time identification of state variables from the biofilter by continuously monitoring several important (easily measurable) parameters such as, inlet pollutant concentrations (using a gas chromatograph), gas flow rate (using a mass flow controller), humidity (using relative humidity sensors), filter bed pH, and temperature (using appropriate sensors). Real-time prediction of pollutant RE is then possible, wherein the acquired data (after proper noise filtering) is continuously integrated to an existing database of information (model inputs and outputs) and the ANN model can then be trained in either online or offline mode. Although, ANNs have found widespread application in real-time control of different industrial (chemical) processes and wastewater treatment systems, this research area still remains unexplored for the monitoring and real-time control of waste-gas treatment systems.

## 5. Conclusions

The RE of two individually operated immobilized cell biofilters (ICBs) was modeled using unit flow and inlet concentration as the input parameters. The best network architecture (2-2-1), determined by a trial and error approach showed that, high learning rates (*η*-0.9), low momentum term (*α*-0.3), with a training cycle of 40,000, are favorable conditions for high performance predictions. The developed BPNN model was able to identify all the peaks and plains of the data under different operating conditions with much less error (<15%). High REs (>93.7%) can be consistently maintained in the ICB, if the inlet H_2_S or NH_3_ concentration is maintained at <120 ppmv, at a unit flow of 2 per min, irrespective of the ICB operating volume. Furthermore, the results from this study evoke that neural networks can capture and extract complex relations among the easily measurable parameters, like unit flow and concentration, in an ICB process and forebode the performance in a meaningful manner.

## Figures and Tables

**Table 1:** Basic statistics of the training data set.

Variable	Basic statistics					
	*N*	Mean	Std deviation	Minimum	Maximum	Sum square
Inputs						
Unit flow, per min	102	1.46	0.36	0.93	2.46	148.92
Concentration, ppmv	102	57.92	27.84	10	150	5908

Outputs						
RE, %	102	94.33	9.69	52.5	100	9621.8

**Table 2:** Basic statistics of the test data set.

Variable	Basic statistics					
	*N*	Mean	Std deviation	Minimum	Maximum	Sum square
Inputs						
Unit flow, per min	32	1.44	0.33	0.92	2.46	46.15
Concentration, ppmv	32	61	27.01	12	150	1952

Outputs						
RE, %	32	94.32	7.31	66.8	100	3018.1

**Table 3:** Network training parameters for choosing the best network architecture.

Training parameters	Range of values	Best value
Training cycle	1000–40000	40000
Number of neurons in input layer	2	2
Number of neurons in hidden layer	2–8	2
Number of neurons in output layer	1	1
Learning rate	0.1–0.9	0.9
Momentum term	0.1–0.9	0.3

Fixed parameters during training		
Error tolerance	0.0001	
Epoch size	25	
Training algorithm	Standard BEP	
Number of training data set	102	
Number of test data set	32	
*R* ^2^ training	0.8716	
*R* ^2^ testing	0.8484	

**Table:** (a) Input to hidden layer weights

	*W*_11_	*W*_12_
Unit flow, per min	−6.61	−8.00
Concentration, ppmv	2.49	−26.6
*Bias term *	−8.19	1.95

*W*_11_,  *W*_12_: Weights between neurons in input layer and hidden layer.

**Table:** (b) Hidden to output layer weights

	RE, %
*W*_21_	1.56
*W*_22_	2.28
*Bias term *	−1.03

*W*_21_, *W*_22_: Weights between neurons in hidden layer and output layer.

**Table 5:** Sensitivity analysis of inputs for the trained network.

Parameters	Absolute average sensitivity, AAS
	RE, %
Unit flow, per min	0.5628
Concentration, ppmv	0.4371

## Abbreviations, Symbols, and Nomenclature

AAS: Absolute average sensitivity
AI: Artificial intelligence
ANN: Artificial neural network
BPNN: Back propagation neural network
BEP: Back error propagation
EC: Elimination capacity, g/m^3^ · h
ICBs: Immobilized cell biofilters
MLP: Multi layered perceptron
RE: Removal efficiency, %
C/N: Carbon to nitrogen ratio
*R*^2^: Regression coefficient
*W*_*ij*_: Connection weights between layers
*θ*_*ij*_: Bias terms
*X*_1_, *X*_2_: Inputs to the neural network model
*Y*_1_: Output from the neural network model
*N*_*Tr*⁡_: Number of data points in the training data set
*N*_Te_: Number of data points in the test data set
*N*: Number of cases analyzed
*η*: Learning rate
*α*: Momentum term
*T*_*c*_: Training cycle
*N*_*I*_: Number of neurons in the input layer
*N*_*O*_: Number of neurons in the output layer
*N*_*H*_: Number of neurons in the hidden layer
RE_exp⁡_: Experimental removal efficiency, %
RE_pred_: Predicted removal efficiency, %.
